# Experimental field tests of Batesian mimicry in the swallowtail butterfly *Papilio polytes*

**DOI:** 10.1002/ece3.4207

**Published:** 2018-07-12

**Authors:** Daniela H. Palmer, Yue Qian Tan, Susan D. Finkbeiner, Adriana D. Briscoe, Antónia Monteiro, Marcus R. Kronforst

**Affiliations:** ^1^ Committee on Evolutionary Biology University of Chicago Chicago Illinois; ^2^ Department of Ecology and Evolution University of Chicago Chicago Illinois; ^3^ Department of Biological Sciences National University of Singapore Singapore Singapore; ^4^ Department of Ecology and Evolutionary Biology University of California Irvine California

**Keywords:** Batesian mimicry, polymorphism, sexual dimorphism, wing pattern

## Abstract

The swallowtail butterfly *Papilio polytes* is known for its striking resemblance in wing pattern to the toxic butterfly *Pachliopta aristolochiae* and is a focal system for the study of mimicry evolution. *Papilio polytes* females are polymorphic in wing pattern, with mimetic and nonmimetic forms, while males are monomorphic and nonmimetic. Past work invokes selection for mimicry as the driving force behind wing pattern evolution in *P. polytes*. However, the mimetic relationship between *P. polytes* and *P. aristolochiae* is not well understood. In order to test the mimicry hypothesis, we constructed paper replicas of mimetic and nonmimetic *P. polytes* and *P. aristolochiae*, placed them in their natural habitat, and measured bird predation on replicas. In initial trials with stationary replicas and plasticine bodies, overall predation was low and we found no differences in predation between replica types. In later trials with replicas mounted on springs and with live mealworms standing in for the butterfly's body, we found less predation on mimetic *P. polytes* replicas compared to nonmimetic *P. polytes* replicas, consistent with the predator avoidance benefits of mimicry. While our results are mixed, they generally lend support to the mimicry hypothesis as well as the idea that behavioral differences between the sexes contributed to the evolution of sexually dimorphic mimicry.

## 1 INTRODUCTION

Mimicry is a classic example of evolution by natural selection. Bates (1862) noted the resemblance between sympatric but distantly related butterfly species, some of which were unpalatable to predators and some of which were palatable. Bates posited that if predators sampled the unpalatable species first, they would subsequently avoid this species and its palatable mimic. Batesian mimicry thus relies on the interactions between the warning signal of the unpalatable species, the predators that avoid the warning signal, and the mimic's ability to dupe the predator by emulating the warning signal (Ruxton, Sherratt, & Speed, 2004).

How predators perceive and respond to warning phenotypes are consequential questions for understanding how natural selection shapes mimetic adaptations. Batesian mimicry is known to occur across animals and plants through different sensory modalities, but visual signals are the best known and understood (Cott, 1940). Visual mimicry signals include changes in coloration, body structure, and behavior, and these often function in concert to achieve integrated mimetic phenotypes (Wickler, 1968). Birds are a major predator class across invertebrate and vertebrate Batesian mimicry systems (Cott, 1940; Wickler, 1968) and have become models for studying predator psychology and visual physiology. Depending on the mimicry system, birds can learn to avoid mimetic phenotypes (Endler, 1991; Gittleman & Harvey, 1980; Guilford, 1990; Roper & Redston, 1987), or may evolve innate avoidance if models are harmful enough (Schuler & Hesse, 1985; Smith, 1975, 1977).

*Papilio polytes* is a female‐limited polymorphic mimic distributed widely throughout Southeast Asia; males have a single nonmimetic phenotype and females have either a male‐like nonmimetic form or one of three mimetic forms that each resembles a distinct toxic *Pachliopta* swallowtail. A rich body of theory and experimental work addressing the evolutionary genetics of mimicry and mate choice preferences has emerged from this system (Clarke & Sheppard, 1972; Fryer, 1914 Charlesworth & Charlesworth, 1975; Kunte 2009; Kitamura & Imafuku, 2010, 2015; Low & Monteiro, 2018; Nishikawa et al., 2013; Ohsaki, 1995; Uésugi, 1996) including the discovery of *doublesex* as the genetic locus controlling mimetic polymorphism in *P. polytes* (Kunte et al., 2014; Nishikawa et al., 2015). While this work has clarified the molecular underpinnings of *P. polytes* and *Pachliopta* mimicry, less has been done experimentally to examine the ecological consequences associated with the mimetic relationship itself.

Mimicry in *P. polytes* was first reported by naturalists who noted the similarity in wing patterns between morphs of *P. polytes* and two *Pachliopta* species (Fryer, 1913; Wallace, 1865). Clarke and Sheppard (1972) later found correlations between the ranges of different mimetic morphs and their putative corresponding models across Southeast Asia. Ohsaki (1995) examined beak marks on the wings of wild‐caught *P. polytes* and *Pachliopta aristolochiae* as a proxy for bird predation. His analysis reported comparable beak mark percentages (28%–29%) on the toxic *P. aristolochiae* and the mimetic form of *P. polytes* that matches the *P. aristolochiae* wing pattern. In contrast, Ohsaki found an elevated percentage of beak marks (53%) on nonmimetic *P. polytes*, suggesting that nonmimetic *P. polytes* experienced more predation than mimetic *P. polytes* females. Uésugi (1996) conducted feeding trials with seven wild‐caught birds (*Hypsipites amaurotis*) in a laboratory setting and found that after an initial training session where birds were fed *P. aristolochiae* the birds reduced their consumption of mimetic *P. polytes*, consistent with a learned aversion to the wing pattern. In addition, analyses of flight kinematics suggest that mimicry extends beyond wing patterning to behavioral similarity between *P. polytes* and *P. aristolochiae* in wing movements and flight path (Kitamura & Imafuku, 2010, 2015). Taken together, these studies are highly suggestive of an adaptive resemblance between mimetic *P. polytes* and *P. aristolochiae* resulting in predator avoidance, but there exist no direct tests of Batesian mimicry using natural populations of these species and free‐ranging bird predators.

Replicas of naturally occurring prey have been used to measure predator‐mediated natural selection in diverse taxa, including insects (Lövei & Ferrante, 2017), fish (Caley & Schluter, 2003), frogs (Saporito, Zuercher, Roberts, Gerow, & Donnelly, 2007), salamanders (Kuchta, 2005), lizards (Stuart‐Fox, Moussalli, Marshall, & Owens, 2003), snakes (Pfennig, Harcombe, & Pfennig, 2001), turtles (Marchand, Litvaitis, Maier, & DeGraaf, 2002), birds (Ibáñez‐Álamo et al., 2015), and mice (Vignieri, Larson, & Hoekstra, 2010). These experiments allow for precise manipulation of artificial models in order to test specific hypotheses about how mimicry phenotypes, or parts thereof, may experience differential predation. The artificial prey method has been implemented in diverse butterfly systems to address the relationship between wing patterning and predation (Dell'Aglio, Stevens, & Jiggins, 2016; Finkbeiner, Briscoe, & Mullen, 2017; Finkbeiner, Briscoe, & Reed, 2012, 2014; Finkbeiner, Fishman, Osorio, & Briscoe, 2017; Ho, Schachat, Piel, & Monteiro, 2016; Merrill et al., 2012; Seymoure & Aiello, 2015; Wee & Monteiro, 2017). In this study, we applied the artificial prey method to study how female‐limited Batesian mimicry operates in wild populations. We constructed replicas of *P. polytes* morphs and *P. aristolochiae,* with realistic color patterns and reflectance, and exposed them to natural predators in the field. We directly assayed bird predation rates based on attack marks or the loss of a bait and tested for differential predation among the sexes and morphs of *P. polytes* in comparison with *P. aristolochiae* in order to analyze the selective advantage of mimicry.

## 2 MATERIALS AND METHODS

We conducted two complementary predation experiments to address different aspects of mimicry in the field. In the first phase, we deployed a large number of stationary artificial prey of four different phenotypes to assay the role of wing patterning alone. Phase I replicas were deployed in an open wing basking position. In the second phase, we used fewer artificial prey types but incorporated biologically meaningful features like butterfly replica movement and a live bait providing body movement and odor to make the trials more realistic. Phase II replicas used a closed wing resting position.

### 2.1 Phase I

#### 2.1.1 Paper replica construction

**Figure 1:**
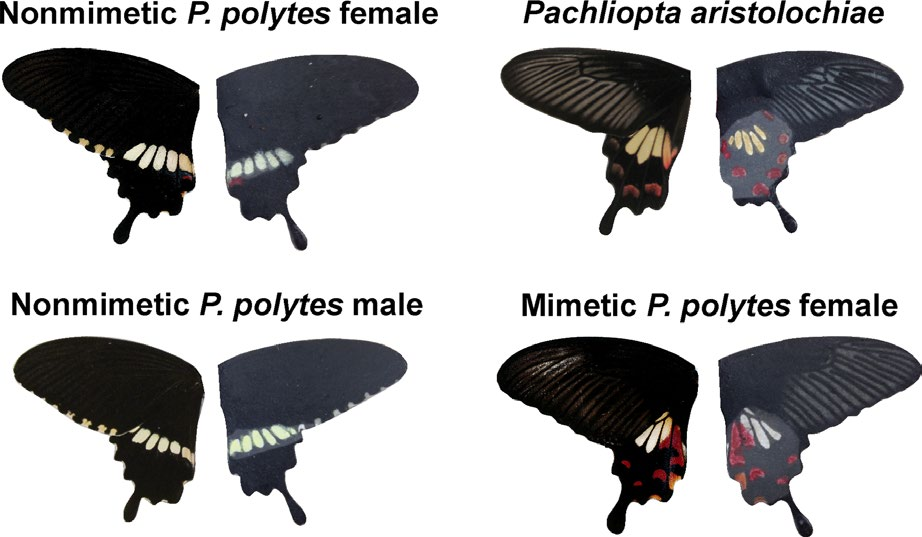
Hypothesized Batesian mimicry between *Papilio polytes* and *Pachliopta aristolochiae*. For each panel, left depicts real wing, and right depicts Phase I wing replica

We constructed four artificial prey types: mimetic *Papilio polytes* female, nonmimetic *P. polytes* female, *P. polytes* male (which are always nonmimetic), and *Pachliopta aristolochiae* (Figure 1). *P. aristolochiae* represents the toxic, unpalatable model in this mimicry system whereas the *Papilio* morphs are nontoxic and palatable. Although *P. polytes* has multiple mimetic forms across its range, we built our replicas based on the local mimetic morph found in Singapore, where all experimental trials were conducted. Similarly, we based our *P. aristolochiae* replicas on the local morph that *P. polytes* mimics. Artificial butterfly replicas were designed following methods described in Finkbeiner et al. (2012), where natural butterfly wings were referenced to create spectrally matched paper wings. We first generated digital images of each replica type's wing pattern in Adobe Illustrator and then printed the wings on low‐reflective Whatman qualitative filter paper (No. 1001‐917), using an Epson Stylus Pro 4880 printer with UltraChrome K3 ink. We applied Crayola^®^ crayon (Gel FX yellow) over the white/yellow hindwing patches of the printed wings where reflectance properties were difficult to reproduce with printed colors alone. We cut out the printed wings with a laser cutter and affixed them to black cardstock backings. To increase the replicas’ durability in the field we dipped the nonchromatic (black/gray) portions of the replicas in molten paraffin wax. Finally, we attached plasticine abdomens (Newplast^®^) to capture the imprints left by predators. The three *Papilio* replica types were given black abdomens and the *Pachliopta* replicas had half black, half red abdomens to emulate the abdomens of real *Pachliopta*.

To evaluate the similarity between real butterflies and our constructed replicas we measured the reflectance spectra of real butterfly wings and abdomens, and artificial butterfly wings (after the molten wax treatment) and abdomens using an Ocean Optics USB2000 fiber optic spectrometer, with a bifurcating fiber cable (R400‐7‐UV‐vis, Ocean Optics) connected to a deuterium–halogen tungsten lamp (DH‐2000, Ocean Optics). We used a white spectralon standard (WS‐1‐SL, Labsphere) during calibration, and placed the detecting fiber in a probe holder at a 45° angle to the plane of the butterfly wing. We designed butterfly replicas to resemble butterflies basking with wings open, and therefore only utilized the dorsal side.

#### 2.1.2 Predation tests

**Figure 2:**
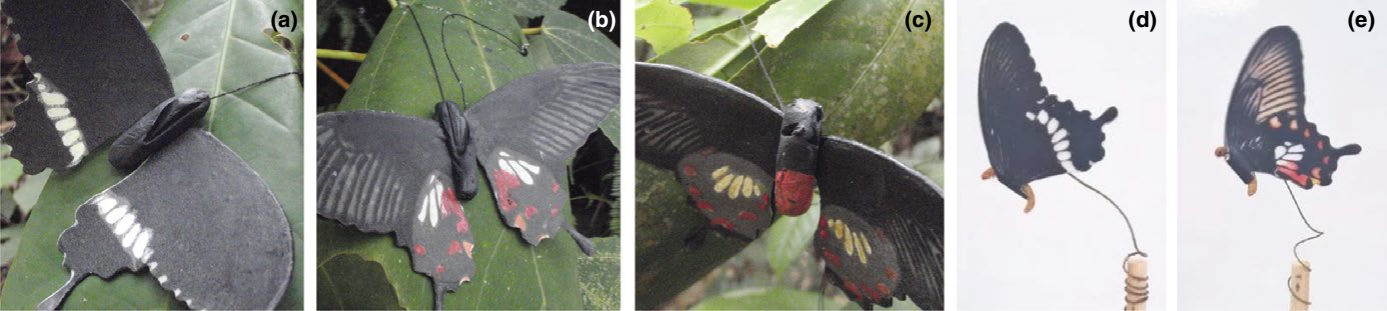
Fully assembled butterfly replicas. (a–c) Phase I replicas showing bird beak marks on the plasticine bodies. (d–e) Phase II replicas with mealworm bodies mounted on wooden sticks

All experimental data were collected in Singapore between June and July 2014. The three field sites were Kent Ridge Park, MacRitchie Reservoir, and Pulau Ubin. A total of 192 paper replicas were tested at Kent Ridge Park, 384 were tested at MacRitchie Reservoir, and 1,216 were tested at Pulau Ubin due to size differences in the available area of the field sites. Kent Ridge Park and Pulau Ubin both contain fragmented forest, and MacRitchie Reservoir contains mature forest. Jain, Lim, and Webb (2017) reported relative abundances of *P. aristolochiae* to *P. polytes* of approximately 1:10 for fragmented forest and 1:17 for mature forest. Replicas were placed in the field in sets of 16 individuals (4 of each type), with at least a 2 m distance between individual paper butterflies. These were attached to plants at a height of approximately 1.5 m with either thread or Blu‐Tack. Each set of 16 was spaced at least 200 m apart from the next to reduce the effects of predator learning on predation attempts, and each set locality was only used once. Replicas remained in place for 4 days and were examined every day for predation marks. Attacks were recorded when beak marks were found imprinted on the plasticine body (Figure 2a–c). Other markings on the plasticine such as insect mandibular imprints were not counted in these attacks. To analyze attack data, we used a generalized linear model (GZLM) with a binomial distribution (replica attacked or not), and replica type and site identity as fixed effects.

### 2.2 Phase II

#### 2.2.1 Selection of field sites

Four new field sites were identified, Jurong Eco Garden, Medicinal Garden at Khoo Teck Puat Hospital, North Buona Vista Road, Singapore (01°29′N, 103°78′E) and the degrading secondary forest along Upper Aljunied Road (01°34′N, 103°87′E), in order to avoid repeated interactions with predators involved in previous trials. All sites consist of fragmented forest, but the North Buona Vista Road site and Upper Aljunied Road sites were reported to have no *P. aristolochiae* sightings nor *P. aristolochiae* host plants (Y.Q. Tan, personal observation). We conducted preliminary trials at the new sites to ensure that birds were actively seeking prey at these locales. We constructed paper replicas of *Mycalesis perseus*, a common, palatable species in Singapore (Ho et al., 2016) and placed 10 replicas in 2 m intervals at each site. A site was considered to experience active predation if 50% of the replicas were attacked within 2 days. All four new sites (JEG, KTPH, NBVR, UAR) fulfilled this condition and were used in subsequent trials.

#### 2.2.2 Paper replica construction

We constructed two new artificial prey types to resemble mimetic and nonmimetic *P. polytes* with their wings closed, showing only their ventral wing surfaces. We generated digital images of these wings using a Leica DMS1000 and Adobe Photoshop CS6. The wing images were printed on Daler‐Rowney 95gsm A4 Sketch Paper using a Samsung Laser Jet 500 Colour M551 printer. The printed white patches were colored using a Derwent Studio Chinese White 72 color pencil to more closely match the real specimens. We treated the nonprinted side of the wings with colorless paraffin wax to waterproof them and capture potential beak imprints without altering the printed colors. Reflectance spectra were taken from ventral wing surfaces of real *P. polytes* and from the printed replicas using an Ocean Optics USB2000 spectrometer and using a light probe placed at 90° to the sample. One live mealworm (*Tenebrio molitor* larva) was secured to each pair of wings using Faber‐Castell Tack‐It and these were mounted on wooden sticks using green floral wire (Figure 2d–e). We coiled the mounting wire to enable wind‐driven movement of the replicas. The base of each wooden stick was coated with a water‐based insect repellent to deter unwanted (nonavian) predators.

#### 2.2.3 Predation tests

At each of the sites (JEG, KTPH, NBVR, UAR) we set out three groups of 10 *P. polytes* replicas (five mimetic and five nonmimetic). Within each group, alternating mimics and nonmimics were planted in the ground at a height of 25–30 cm, approximately 2 m apart from one another. Replicas remained at the site for 2 days for each trial. These were inspected daily and predation was recorded when the mealworm was removed or damaged or when bite marks were present on the wings. We used a GZLM with a binomial distribution, and replica type and site identity as fixed effects to analyze differences in predation.

### 2.3 Discriminability tests

We calculated discriminability using a bird vision model to verify that our paper replicas accurately resembled their real counterparts through the eyes of avian predators. A number of insectivorous birds inhabit Singapore (Castelletta, Thiollay, & Sodhi, 2005), but the specific predators of *P. polytes* in this area are unknown. For each replica type, we compared color patches (white, black, and red if present) between real specimens and paper replicas and calculated their similarity in units of just noticeable differences (JNDs) using the receptor‐noise model of Vorobyev and Osorio (1998). The comparisons were made using the blue tit (*Cyanistes caeruleus*) cone sensitivities, which represent the UV‐type avian visual system. We followed the work of Hart, Partridge, Cuthill, and Bennett (2000) including the effects of blue tit ocular media and used a Weber fraction = 0.05 and relative abundances of cones (UV = 0.37, *S* = 0.7, *M* = 0.99, *L* = 1). We analyzed replicas from Phase I using low light intensity and Endler's (1993) forest shade irradiance spectra because they were placed in the forest understory. For Phase II replicas we used low light intensity and Endler's (1993) daylight irradiance spectra because these replicas were placed in open areas. Low light intensity conditions were selected for both Phase I and Phase II as a more ecologically relevant prediction: avian predators are most active during morning hours when natural light is less bright, and previous work in the tropics has shown experimental evidence that avian attacks on artificial butterfly models occur almost exclusively during the early morning (Finkbeiner et al., 2012).

## 3 RESULTS

### 3.1 Discriminability tests

**Figure 3:**
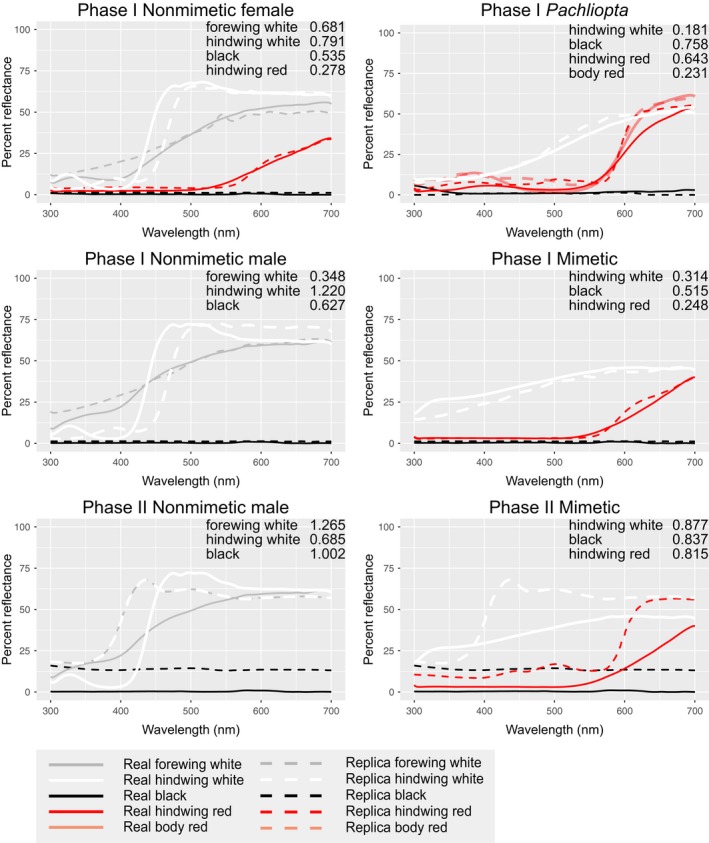
Reflectance spectra of real and replica color patches, and results of discriminability modeling of the UV‐type avian visual system. Percent reflectance for each color patch is shown from 300 to 700 nm, which is the avian visual range. Avian vision modeling results for each color patch are in units of just noticeable differences (JNDs)

The spectral comparisons between color patches of real specimens and paper replicas are presented in Figure 3, showing that the reflectance spectra are generally concordant between the real specimens and replicas. The avian vision modeling results showed that 17 out of 20 color patch comparisons had JND values below the discriminability threshold of 1.0, and were thus considered indiscriminable to birds, while the other three comparisons still had low JND values between 1.0 and 1.3.

### 3.2 Phase I

A total of 1,792 individual paper replicas were tested among the three sites (192 at Kent Ridge Park, 384 at MacRitchie Reservoir, and 1,216 at Pulau Ubin). We observed a variety of markings on the plasticine bodies of the replicas including imprints from bird beaks (Figure 2a–c), arthropod mandibles, and from one small reptile or mammal. Across the four types, we calculated an overall avian predation rate of 1.9%. Cases where the paper replicas could not be found at the end of the test period were excluded from the data set.

Avian predation results for Phase I are summarized in Table 1. While we did find a larger number of attacks on nonmimetic female *P. polytes* compared to mimetic *P. polytes,* consistent with the expectations of Batesian mimicry, we also found that *P. aristolochiae* replicas experienced the most attacks and nonmimetic males the fewest attacks. Results from our GZLM showed that replica type was not a significant predictor of attack number, but site identity was a significant predictor (Table 2). A subsequent model including only site identity as a fixed effect showed that replicas at the Pulau Ubin site experienced significantly less predation than Kent Ridge Park or MacRitchie Reservoir (Table 2).

**Table 1:** Avian predation in Phase I experiments. Each cell shows the number of replicas attacked and the number of replicas not attacked in parentheses

	*Papilio polytes* Nonmimetic female	*P. polytes* Nonmimetic male	*P. polytes* Mimetic	*Pachliopta aristolochiae*
Kent Ridge Park	3 (46)	1 (47)	1 (48)	3 (45)
MacRitchie Reservoir Park	3 (90)	2 (90)	1 (87)	5 (83)
Pulau Ubin	3 (279)	1 (276)	4 (274)	5 (278)
Total	9 (415)	4 (413)	6 (409)	13 (406)

**Table 2:** Results of generalized linear model for Phase I experiments

Model	Estimate	*SE*	*z*‐Value	*p*‐Value	*df*
Predation ~ replica type + site identity					
Replica type	0.2075	0.1629	1.273	0.202854	1,791
Site identity	−0.7213	0.2156	−3.345	0.000822	1,791
Predation ~ site identity					
Kent Ridge Park vs. MacRitchie Reservoir	−0.3882	0.4733	−0.820	0.41213	1,791
Kent Ridge Park vs. Pulau Ubin	−1.3921	0.4563	−3.051	0.00228	1,791
MacRitchie Reservoir vs. Pulau Ubin	1.0039	0.4139	2.425	0.01529	1,791

### 3.3 Phase II

A total of 120 individual paper replicas (mimetic and nonmimetic *P. polytes*) were tested among the four sites (15 mimetic and 15 nonmimetic at each site). Predation results for Phase II are summarized in Table 3. We again tested for differential predation between mimetic and nonmimetic types. At each site mimics experienced fewer predation events than nonmimics, and all attacks combined showed approximately 30% less predation on mimics than nonmimics. However, the *p*‐value for replica type was slightly above the statistical significance cutoff of 0.05 (Table 4). Predation was not significantly different between sites in Phase II (Table 4).

**Table 3:** Avian predation in Phase II experiments. Each cell shows the number of replicas attacked and the number of replicas not attacked in parentheses

	*Papilio polytes* Nonmimetic	*Papilio polytes* Mimetic
Jurong Eco Garden	8 (7)	5 (10)
Khoo Teck Puat Hospital	9 (6)	6 (9)
North Buona Vista Road	8 (7)	5 (10)
Upper Aljunied Road	9 (6)	8 (7)
Total	34 (26)	24 (36)

**Table 4:** Results of generalized linear model for Phase II experiments

Model	Estimate	*SE*	*z*‐Value	*p*‐Value	*df*
Predation ~ replica type + site identity					
Replica type	−0.67768	0.37175	−1.823	0.0683	119
Site identity	0.13775	0.16653	0.827	0.4081	119

## 4 DISCUSSION

We generated realistic artificial prey and tested for mimetic protection from predation in *P. polytes*. In Phase I, we observed generally low attack rates and did not find significant differences in predation between mimetic and nonmimetic *P. polytes*. In Phase II, we observed the expected lower levels of predation on mimetic replicas compared to nonmimetic replicas at individual sites, but these were not statistically significant overall and fell slightly above the significance value of 0.05. These results suggest that mimetic morphs of *P. polytes* experience a selective advantage over nonmimetic *P. polytes* by deterring predators, consistent with the basic predictions of Batesian mimicry.

Trials in Phase I resulted in significantly lower predation rates in comparison with Phase II (1.91% and 48.33% respectively; *χ* ^2^ = 497.01, *df* = 1, *p* < 0.001). Phase I predation rates were also lower than those reported in other butterfly predation studies using similar methods (6.38% in Finkbeiner et al., 2012; 54.4% in Ho et al., 2016; 51% in Wee & Monteiro, 2017). Replicas in Phase I were fixed to leaves and remained stationary while those in Phase II were mounted on springs that allowed for movement and also incorporated a live bait. It is possible that the immobile conditions of Phase I made the artificial prey difficult for predators to visually detect. Other possible explanations for differences in predation rates between field sites might include the availability of alternative prey, frequency of models to mimics, predator abundance, and/or predator/prey seasonality (Finkbeiner et al., 2018).

In Phase II, we observed slightly increased attacks on nonmimetic morphs compared to mimetic morphs at each individual site that just missed the *p*‐value significance cutoff of 0.05, when analyzed using a GZLM. This result suggests a modest benefit to mimetic individuals of this species, and with higher power and a larger sample size, a significant difference in predation is expected between mimetic and nonmimetic individuals. More trials need to be conducted to explore this question. The black reflectance values in Phase II replicas were higher than in Phase I, possibly making the Phase II replicas more detectable to predators and thus more likely to be attacked overall. Although Phase I replicas more closely resembled their real counterparts than Phase II replicas (Phase I had lower JNDs overall), Phase II replicas elicited predation differences in the direction expected under Batesian mimicry.

Behavioral differences between the sexes have long been hypothesized to drive the evolution of female‐limited mimicry. Wallace (1865) proposed that female butterflies experience more predation than their male counterparts when searching for host plants while laden with eggs and while hovering over plants during oviposition. The evolution of flight mimicry in *P. polytes* supports this idea; wing beat and flight path are significantly different between mimetic and nonmimetic individuals, and mimetic individuals fly like the toxic *Pachliopta aristolochiae* model (Kitamura & Imafuku, 2010, 2015). Ohsaki (1995) proposed that females disproportionately benefit from evolving mimicry and indeed found that wild‐caught nonmimetic *P. polytes* females had nearly double the proportion of beak marks on their wings compared to *P. polytes* males (which are always nonmimetic). More recently, Su, Lim, and Kunte (2015) tested the mimetic resemblance of several sexually monomorphic and female‐limited Batesian mimics, including *P. polytes*, using bird vision models. Their results show that females are better mimics than males of sexually monomorphic taxa, and that female‐limited mimics are as good as sexually monomorphic mimics, supporting the idea that females disproportionately benefit from mimicry. If the benefit to mimicry derives in part from behavioral differences between the sexes, our Phase I results may reflect baseline predation that *P. polytes* experience due to visual detection alone and in the absence of any behavior, including flight behavior and smaller movement such as body/leg/antennal movement as well as in the absence of body odor.

The continuum between Batesian and Müllerian mimicry is determined by the relative unpalatability of the taxa involved (Huheey, 1976). In Batesian systems, where the mimic is palatable, both the abundance and unpalatability of the model are expected to strongly influence the mimic's benefit (Finkbeiner et al., 2018; Lindström, Alatalo, & Mappes, 1997). *Pachliopta aristolochiae* are known to occur at some of the experimental sites, and their relative abundances in comparison with *P. polytes* across those sites are variable (Jain et al., 2017). The degree of unpalatability of *P. aristolochiae* to bird predators is not well known and could influence how strongly birds avoid its phenotype (Speed, 2000). Feeding experiments using captive bulbuls (*Hypsipetes amaurotis*) found that birds experienced a negative physical reaction immediately after ingesting *P. aristolochiae*, and subsequently decreased their feeding on mimetic *P. polytes* (Uésugi, 1996). It is interesting that, however, birds in Uésugi's experiment also showed a small decrease in feeding on nonmimetic *P. polytes* over the course of the feeding trials. This could reflect a generalization of avoidance beyond the mimicry phenotype to, for example, butterflies with black wings (Johki, 1983; Johki, Kon, & Hidaka, 1986; Speed, 2000). It could also imply that *P. polytes* itself may be somewhat unpalatable. Smetacek (2006) presented freshly caught *P. polytes* to free‐ranging predators in Uttaranchal, India and observed that only 44.4% were eaten by birds in comparison with 77%–100% of palatable control species eaten. If reclassified, this would not be the first case in which a Batesian mimic was actually found to be an unpalatable Müllerian comimic (Ritland & Brower, 1991).

Predator psychology is complex and acts as the selective agent in Batesian mimicry systems (Speed, 2000). Over 100 years ago, Fryer (1913) recorded detailed observations of birds chasing and eating various butterfly species, including mimetic and nonmimetic *P. polytes* and distantly related species thought to be noxious to birds. Although we cannot deny the striking resemblance between distantly related species, the notion of mimicry is oversimplified in comparison with the dynamics taking place in nature. Artificial prey experiments have become powerful tools for mimicry research in natural settings by allowing us to manipulate the signals that predators encounter and measure predators’ responses in the field. Significant advances using this method (especially for butterflies) include assessing the influence of roosting behavior on warning signals (Finkbeiner et al., 2012), studying the relative contributions of wing color versus pattern to predator deterrence (Finkbeiner et al., 2014), uncovering how eyespot size and number influence predation (Ho et al., 2016; Stevens, Hardman, & Stubbins, 2008), analyzing the evolution of novel colors in warning signals (Dell'Aglio et al., 2016; Finkbeiner, Fishman, et al., 2017; Wee & Monteiro, 2017), and dissecting the importance of white bands (false boundaries) and disruptive coloration for protection from predators (Seymoure & Aiello, 2015). Further experiments are needed that integrate phenotypic and behavioral qualities of Batesian mimics with predator psychology and that assess the importance of model frequency dependence in Batesian systems. *Papilio* swallowtails are likely to be a key system for these studies, owing to the frequent occurrence of mimicry in this group. With these further studies, the growing literature concerning the genetics of mimicry will also benefit from a better understanding of how natural selection on mimicry proceeds in natural populations.

## CONFLICT OF INTEREST

None declared.

## AUTHOR CONTRIBUTIONS

D.H.P., Y.Q.T., S.D.F., A.M., and M.R.K. designed experiments. D.H.P., Y.Q.T., and S.D.F. collected data. D.H.P., Y.Q.T., S.D.F., and A.D.B. analyzed data. D.H.P. wrote the manuscript. All authors edited and approved the final manuscript.

## References

[ece34207-bib-0001] Bates, H. W. (1862). Contributions to an insect fauna of the Amazon valley. Lepidoptera: Heliconidae. Transactions of the Linnean Society of London, 23, 495–566. 10.1111/j.1096-3642.1860.tb00146.x

[ece34207-bib-0002] Caley, M. J. , & Schluter, D. (2003). Predators favour mimicry in a tropical reef fish. Proceedings of the Royal Society B: Biological Sciences, 270, 667–672. 10.1098/rspb.2002.2263 12713739 PMC1691296

[ece34207-bib-0003] Castelletta, M. , Thiollay, J. M. , & Sodhi, N. S. (2005). The effects of extreme forest fragmentation on the bird community of Singapore Island. Biological Conservation, 121, 135–155. 10.1016/j.biocon.2004.03.033

[ece34207-bib-0005] Charlesworth, D. , & Charlesworth, B. (1975). Theoretical genetics of Batesian mimicry II. Evolution of supergenes. Journal of Theoretical Biology, 55, 305–324. 10.1016/S0022-5193(75)80082-8 1207161

[ece34207-bib-0006] Clarke, C. A. , & Sheppard, P. M. (1972). The genetics of the mimetic butterfly *Papilio polytes* L. Philosophical Transactions of the Royal Society of London. Series B, Biological sciences, 263, 431–458. 10.1098/rstb.1972.0006 4402450

[ece34207-bib-0007] Cott, H. B. (1940). Adaptive coloration in animals (pp. 292–294). London, UK: Methuen.

[ece34207-bib-0008] Dell'Aglio, D. D. , Stevens, M. , & Jiggins, C. D. (2016). Avoidance of an aposematically coloured butterfly by wild birds in a tropical forest. Ecological Entomology, 41, 627–632. 10.1111/een.12335 27708481 PMC5026159

[ece34207-bib-0009] Endler, J. A. (1991). Interactions between predators and prey In Krebs J. R., & Davies N. B. (Eds.), Behavioural Ecology, 3rd ed. (pp. 169–196). Oxford, UK: Blackwell.

[ece34207-bib-0010] Endler, J. A. (1993). The color of light in forests and its implications. Ecological Monographs, 63, 1–27. 10.2307/2937121

[ece34207-bib-0011] Finkbeiner, S. D. , Briscoe, A. D. , & Mullen, S. P. (2017). Complex dynamics underlie the evolution of imperfect wing pattern convergence in butterflies. Evolution, 71, 949–959. 10.1111/evo.13165 28052323

[ece34207-bib-0012] Finkbeiner, S. D. , Briscoe, A. D. , & Reed, R. D. (2012). The benefit of being a social butterfly: Communal roosting deters predation. Proceedings of the Royal Society B: Biological Sciences, 279, 2769–2776. 10.1098/rspb.2012.0203 22438492 PMC3367783

[ece34207-bib-0013] Finkbeiner, S. D. , Briscoe, A. D. , & Reed, R. D. (2014). Warning signals are seductive: Relative contributions of color and pattern to predator avoidance and mate attraction in *Heliconius* butterflies. Evolution, 68, 3410–3420. 10.1111/evo.12524 25200939

[ece34207-bib-0014] Finkbeiner, S. D. , Fishman, D. A. , Osorio, D. , & Briscoe, A. D. (2017). Ultraviolet and yellow reflectance but not fluorescence is important for visual discrimination of conspecifics by *Heliconius erato* . Journal of Experimental Biology, 220, 1267–1276. 10.1242/jeb.153593 28108668

[ece34207-bib-0015] Finkbeiner, S. D. , Salazar, P. , Nogales, S. , Rush, C. , Briscoe, A. D. , Hill, R. I. , … Mullen, S. P. (2018). Frequency‐dependence shapes the adaptive landscape of imperfect Batesian mimicry. Proceedings of the Royal Society of London. Series B, Biological sciences, 285: 20172786 10.1098/rspb.2017.2786 29618547 PMC5904311

[ece34207-bib-0016] Fryer, J. (1913). Field‐observations on the enemies of butterflies in Ceylon. Proceedings of the Zoological Society of London, 83, 613–619.

[ece34207-bib-0017] Fryer, J. C. F. (1914). An investigation by pedigree breeding into the polymorphism of *Papilio polytes*, L. Philosophical Transactions of the Royal Society B: Biological Sciences, 204, 227–254. 10.1098/rstb.1914.0007

[ece34207-bib-0018] Gittleman, J. L. , & Harvey, P. H. (1980). Why are distasteful prey not cryptic? Nature, 286, 149–150. 10.1038/286149a0

[ece34207-bib-0019] Guilford, T. (1990). The evolution of aposematism In Evans D. L., & Schmidt J. O. (Eds.), Insect Defenses: Adaptive Mechanisms and Strategies of Prey and Predators (pp. 23–62). Albany: State University of New York Press.

[ece34207-bib-0020] Hart, N. S. , Partridge, J. C. , Cuthill, I. C. , & Bennett, A. T. D. (2000). Visual pigments, oil droplets, ocular media and cone photoreceptor distribution in two species of passerine bird: The blue tit (*Parus caeruleus* L.) and the blackbird (*Turdus merula* L.). Journal of Comparative Physiology A, 186, 375–387. 10.1007/s003590050437 10798725

[ece34207-bib-0021] Ho, S. , Schachat, S. R. , Piel, W. H. , & Monteiro, A. (2016). Attack risk for butterflies changes with eyespot number and size. Royal Society Open Science, 3, 150614 10.1098/rsos.150614 26909190 PMC4736945

[ece34207-bib-0022] Huheey, J. E. (1976). Studies in warning coloration and mimicry. VII. Evolutionary consequences of a Batesian‐Müllerian spectrum: A model for Müllerian mimicry. Evolution, 30, 86–93. 28565050 10.1111/j.1558-5646.1976.tb00884.x

[ece34207-bib-0023] Ibáñez‐Álamo, J. , Magrath, R. D. , Oteyza, J. , Chalfoun, A. , Haff, T. , Schmidt, K. , … Martin, T. (2015). Nest predation research: Recent findings and future perspectives. Journal of Ornithology, 156, 247–262. 10.1007/s10336-015-1207-4

[ece34207-bib-0024] Jain, A. , Lim, F. K. , & Webb, E. L. (2017). Species‐habitat relationships and ecological correlates of butterfly abundance in a transformed tropical landscape. Biotropica, 49(3), 355–364. 10.1111/btp.12435

[ece34207-bib-0025] Johki, Y. (1983). Studies on avoidance of predation by colouration in insects. Doctoral dissertation, Kyoto University, Kyoto.

[ece34207-bib-0026] Johki, Y. , Kon, M. , & Hidaka, T. (1986). Feeding experiments using the red avadavat, *Amandava amandava*, with reference to the effectiveness of Batesian mimicry. Memoirs of the Faculty of Science, Kyoto University (Series of Biology), 11, 29–42.

[ece34207-bib-0027] Kitamura, T. , & Imafuku, M. (2010). Behavioral Batesian mimicry involving intraspecific polymorphism in the butterfly *Papilio polytes* . Zoological Science, 27, 217–221. 10.2108/zsj.27.217 20192689

[ece34207-bib-0028] Kitamura, T. , & Imafuku, M. (2015). Behavioural mimicry in flight path of Batesian intraspecific polymorphic butterfly *Papilio polytes* . Proceedings of the Royal Society B‐Biological Sciences, 282, 20150483 10.1098/rspb.2015.0483 PMC4590450 26041360

[ece34207-bib-0030] Kuchta, S. R. (2005). Experimental support for aposematic coloration in the salamander *Ensatina eschscholtzii xanthoptica*: Implications for mimicry of Pacific newts. Copeia, 2005, 265–271. 10.1643/CH-04-173R

[ece34207-bib-0031] Kunte, K. (2009). The diversity and evolution of Batesian mimicry in *Papilio* swallowtail butterflies. Evolution, 63, 2707–2716. 10.1111/j.1558-5646.2009.00752.x 19552738

[ece34207-bib-0032] Kunte, K. , Zhang, W. , Tenger‐Trolander, A. , Palmer, D. H. , Martin, A. , Reed, R. D. , … Kronforst, M. R. (2014). *doublesex* is a mimicry supergene. Nature, 507, 229–232. 10.1038/nature13112 24598547

[ece34207-bib-0033] Lindström, L. , Alatalo, R. V. , & Mappes, J. (1997). Imperfect Batesian mimicry—The effects of the frequency and the distastefulness of the model. Proceedings of the Royal Society B‐Biological Sciences, 264, 149–153. 10.1098/rspb.1997.0022

[ece34207-bib-0034] Lövei, G. L. , & Ferrante, M. (2017). A review of the sentinel prey method as a way of quantifying invertebrate predation under field conditions. Insect Science, 24, 528–542. 27686246 10.1111/1744-7917.12405

[ece34207-bib-0035] Low, X. H. , & Monteiro, A. (2018). Dorsal forewing white spots of male *Papilio polytes* (Lepidoptera: Papilionidae) not maintained by female mate choice. Journal of Insect Behavior, 31, 29–41.

[ece34207-bib-0036] Marchand, M. N. , Litvaitis, J. A. , Maier, T. J. , & DeGraaf, R. M. (2002). Use of artificial nests to investigate predation on freshwater turtle nests. Wildlife Society Bulletin, 30, 1092–1098.

[ece34207-bib-0038] Merrill, R. M. , Wallbank, R. W. , Bull, V. , Salazar, P. C. , Mallet, J. , Stevens, M. , & Jiggins, C. D. (2012). Disruptive ecological selection on a mating cue. Proceedings of the Royal Society B: Biological Sciences, 279, 4907–4913. 10.1098/rspb.2012.1968 23075843 PMC3497240

[ece34207-bib-0039] Nishikawa, H. , Iga, M. , Yamaguchi, J. , Saito, K. , Kataoka, H. , Suzuki, Y. , … Fujiwara, H. (2013). Molecular basis of wing coloration in a Batesian mimic butterfly. Papilio Polytes. Science Reports, 3, 3184. 10.1038/srep03184 PMC3822385 24212474

[ece34207-bib-0040] Nishikawa, H. , Iijima, T. , Kajitani, R. , Yamaguchi, J. , Ando, T. , Suzuki, Y. , … Hirakawa, H. (2015). A genetic mechanism for female‐limited Batesian mimicry in *Papilio* butterfly. Nature Genetics, 47, 405–409. 10.1038/ng.3241 25751626

[ece34207-bib-0041] Ohsaki, N. (1995). Preferential predation of female butterflies and the evolution of Batesian mimicry. Nature, 378, 173 10.1038/378173a0

[ece34207-bib-0043] Pfennig, D. W. , Harcombe, W. R. , & Pfennig, K. S. (2001). Frequency‐dependent Batesian mimicry. Nature, 410, 323 10.1038/35066628 11268195

[ece34207-bib-0044] Ritland, D. B. , & Brower, L. P. (1991). The viceroy butterfly is not a Batesian mimic. Nature, 350, 497–498. 10.1038/350497a0

[ece34207-bib-0045] Roper, T. , & Redston, S. (1987). Conspicuousness of distasteful prey affects the strength and durability of one‐trial avoidance learning. Animal Behavior, 35, 739–747. 10.1016/S0003-3472(87)80110-0

[ece34207-bib-0046] Ruxton, G. D. , Sherratt, T. N. , & Speed, M. P. (2004). Avoiding attack: The evolutionary ecology of crypsis, warning signals and mimicry (pp. 139–163). Oxford, UK: Oxford University Press 10.1093/acprof:oso/9780198528609.001.0001

[ece34207-bib-0047] Saporito, R. A. , Zuercher, R. , Roberts, M. , Gerow, K. G. , & Donnelly, M. A. (2007). Experimental evidence for aposematism in the dendrobatid poison frog *Oophaga pumilio* . Copeia, 2007, 1006–1011. 10.1643/0045-8511(2007)7[1006:EEFAIT]2.0.CO;2

[ece34207-bib-0048] Schuler, W. , & Hesse, E. (1985). On the function of warning coloration: A black and yellow pattern inhibits prey‐attack by naive domestic chicks. Behavioral Ecology and Sociobiology, 16(3), 249–255. 10.1007/BF00310988

[ece34207-bib-0049] Seymoure, B. M. , & Aiello, A. (2015). Keeping the band together: Evidence for false boundary disruptive coloration in a butterfly. Journal of Evolutionary Biology, 28, 1618–1624. 10.1111/jeb.12681 26109438

[ece34207-bib-0050] Smetacek, P. (2006). Some distasteful Asian Papilioninae (Papilionidae). Journal of the Lepidopterists’ Society, 60, 82.

[ece34207-bib-0051] Smith, S. M. (1975). Innate recognition of coral snake pattern by a possible avian predator. Science, 187, 759–760. 10.1126/science.187.4178.759 17795249

[ece34207-bib-0052] Smith, S. M. (1977). Coral‐snake pattern recognition and stimulus generalisation by naive great kiskadees (Aves: Tyrannidae). Nature, 265, 535 10.1038/265535a0

[ece34207-bib-0053] Speed, M. P. (2000). Warning signals, receiver psychology and predator memory. Animal Behavior, 60, 269–278. 10.1006/anbe.2000.1430 11007635

[ece34207-bib-0054] Stevens, M. , Hardman, C. J. , & Stubbins, C. L. (2008). Conspicuousness, not eye mimicry, makes “eyespots” effective antipredator signals. Behavioral Ecology, 19, 525–531. 10.1093/beheco/arm162

[ece34207-bib-0055] Stuart‐Fox, D. M. , Moussalli, A. , Marshall, N. J. , & Owens, I. P. (2003). Conspicuous males suffer higher predation risk: Visual modelling and experimental evidence from lizards. Animal Behaviour, 66, 541–550. 10.1006/anbe.2003.2235

[ece34207-bib-0056] Su, S. , Lim, M. , & Kunte, K. (2015). Prey from the eyes of predators: Color discriminability of aposematic and mimetic butterflies from an avian visual perspective. Evolution, 69, 2985–2994. 10.1111/evo.12800 26477885

[ece34207-bib-0058] Uésugi, K. (1996). The adaptive significance of Batesian mimicry in the swallowtail butterfly, *Papilio polytes* (Insecta, Papilionidae): Associative learning in a predator. Ethology, 102, 762–775.

[ece34207-bib-0059] Vignieri, S. N. , Larson, J. G. , & Hoekstra, H. E. (2010). The selective advantage of crypsis in mice. Evolution, 64, 2153–2158. 20163447 10.1111/j.1558-5646.2010.00976.x

[ece34207-bib-0060] Vorobyev, M. , & Osorio, D. (1998). Receptor noise as a determinant of colour thresholds. Proceedings of the Royal Society of London, Series B: Biological Sciences, 265, 351–358. 10.1098/rspb.1998.0302 9523436 PMC1688899

[ece34207-bib-0061] Wallace, A. R. (1865). On the phenomena of variation and geographical distribution as illustrated by the Papilionidae of the Malayan region. Transactions of the Linnean Society of London, 25, 1–71. 10.1111/j.1096-3642.1865.tb00178.x

[ece34207-bib-0062] Wee, J. L. Q. , & Monteiro, A. (2017). Yellow and the novel aposematic signal, red, protect *Delias* butterflies from predators. PLoS ONE, 12, e0168243 10.1371/journal.pone.0168243 28060944 PMC5218396

[ece34207-bib-0063] Wickler, W. (1968). Mimicry in plants and animals (pp. 60–110). London, UK: Weidenfeld & Nicolson.

